# Prevalence and predictors of chronic kidney disease among type 2 diabetic patients worldwide, systematic review and meta-analysis

**DOI:** 10.1186/s13098-023-01202-x

**Published:** 2023-11-28

**Authors:** Eneyew Talie Fenta, Habitu Birhan Eshetu, Natnael Kebede, Eyob Ketema Bogale, Amare Zewdie, Tadele Derbew Kassie, Tadele Fentabil Anagaw, Elyas Melaku Mazengia, Sintayehu Shiferaw Gelaw

**Affiliations:** 1 Department of public health, college of medicine and health sciences, Injibara University, Injibara, Ethiopia; 2 Department of Health Promotion and Health Behavior, Institute of Public Health, College of Medicine and Health Sciences, University of Gondar, PO. Box.196, Gondar, Ethiopia; 3 Department of Health Promotion, School of Public Health, College of Medicine and Health Sciences, Wollo University, Dessie, Ethiopia; 4 Health Promotion and Behavioral science department, College of medicine and health science, Bahir Dar, Houston, Ethiopia; 5 Department of Public Health, College of Medicine and Health Science, Wolkite University, Wolkite, Ethiopia; 6 Department of Public Health, College of Medicine and Health Science, Debre Markos university, Debre Markos, Ethiopia

**Keywords:** Chronic kidney disease, Meta-analysis, Type 2 diabetes mellitus

## Abstract

**Background:**

Diabetes is a complicated, chronic condition that requires ongoing medical attention as well as multiple risk-reduction measures beyond glucose control. The prevalence of chronic kidney disease (CKD) is highly variable in different parts of the world due to various environmental, ethnic, socioeconomic, and rural-urban differences. Diabetes is the leading cause of CKD. This study aimed to estimate the global prevalence of CKD and its associated factors among type 2 diabetes(T2DM) patients, provide scientific evidence for a better understanding of the burden of CKD among diabetes mellitus type 2 patients, and design interventional strategies.

**Methods:**

Preferred Reporting Items for Systematic Reviews and Meta-Analysis (PRISMA) checklist guideline was followed for this review and meta-analysis. The electronic databases (Pub Med, Cochrane Library, Google Scholar, and grey literature) were searched to retrieve articles by using keywords. Joanna Briggs Institute Meta-Analysis of Statistics Assessment and Review Instrument was used to assess the quality of studies. The meta-analysis was conducted using STATA 17 software. The Meta logistic regression was computed to present the pooled prevalence and Odds ratio (OR) of the determinate factors with a 95% confidence interval (CI).

**Results:**

In this systematic review and meta-analysis 20 studies were done in 13 different countries. The pooled magnitude of chronic kidney disease among type 2 DM patients was 27% (95% CI 21%, 33%). The prevalence of chronic kidney disease differs across countries, with the maximum in the USA and the lowest in the United Arab Emirates. Patients with CKD have an elevated risk of severe renal and cardiovascular morbidity and mortality. Renin-angiotensin system inhibitors, sodium-glucose cotransporter-2 inhibitors, glucagon-like peptide 1 receptor agonists, and, more recently, non-steroidal mineralocorticoid receptor antagonists are among the medications that have been demonstrated to slow the progression of CKD. In this systematic review and meta-analysis increased age, obesity, having a history of type 2 diabetes mellitus, smoking history, presence of hypertension, and cardiac heart disease were factors significantly associated with the presence of chronic kidney disease among type 2 diabetic patients.

**Conclusions:**

The prevalence of chronic kidney disease among type 2 diabetes mellitus patients was high based on the included 20 articles. The review reported that old age, hypertension, cardiac disease, smoking, obesity, and duration of diabetes mellitus was predictor variable for chronic kidney disease among type 2 diabetic patients. Therefore, in order to lower the morbidity and mortality from chronic kidney disease among type 2 diabetic patients, it is advised to develop both preventive and curative intervention strategies, such as raising awareness, creating a supportive environment, and prescribing appropriate medication at an early stage.

## Introduction

Diabetes mellitus (DM) is a metabolic systemic multifactorial disease, where the body becomes unable to utilize or produce a sufficient amount of insulin to match the body’s needs [1, 2]. Diabetes is a complicated, chronic condition that need for ongoing medical attention as well as multiple risk-reduction measures beyond glucose control [3, 4]. The World Health Organization(WHO) placed the global targets for diabetes mellitus by 2030, 80% of people living with diabetes mellitus are diagnosed, and that 80% of those living with diabetes mellitus will have good control of glycaemia and blood pressure [5].

Globally, about one in eleven adults have diabetes mellitus, from this 90% have type 2 diabetes mellitus. The main drivers of the T2DM epidemic are the global rise in obesity, sedentary lifestyles, high caloric diets and population aging, which have quadrupled the incidence and prevalence of T2DM [6–8].The estimated global prevalence of diabetes in 2015 was 415 million; by 2040, it is expected to rise to 642 million, with more increase in low- and middle-income nations [9]. DM patients have uncontrolled blood sugar levels and a longer duration of diabetes were considered as independent risk factors of CKD [10]. Diabetes can cause serious health problems, such as heart disease, stroke, and eye and foot problems. Prediabetes also can cause health problems. The good news is that type 2 diabetes can be delayed or even prevented [11].

The studies showed that diabetic individuals develop CKD and exposed to renal complications with considerable morbidity, poor quality of life, and increased healthcare costs [12–14]. The study in Europe stated that the prevalence of CKD in patients with diabetes is expected to increase within increase in cost of treatment [15]. The prevalence of CKD is highly variable in different parts of the world, due to various environmental, ethnic, socioeconomical, and rural-urban variations [16]. In the 2015 Global Burden of Disease Study, kidney disease was the 12th most common cause of death, accounting for 1.1 million deaths worldwide. Overall CKD mortality has increased by 31.7% over the last 10 years, making it one of the fastest rising major causes of death, alongside diabetes and dementia [17].

Chronic kidney disease (CKD) is the abnormalities of kidney structure or function that are present for more than 3 months characterized by low estimated glomerular filtration rate (eGFR) based on serum creatinine measurements. It is one of the complications among T2DM patients [12, 18]. The primary issue for individuals in stages 1 and 2 was that it was asymptomatic at first; metabolic abnormalities were apparent in stages 3 through 5. Diabetic kidney disease (CKD) and other vascular disorders are the main reasons. Estimating the glomerular filtration rate (eGFR), which evaluates kidney function, and finding albuminuria, a sign of kidney damage, are the first steps in diagnosing chronic kidney disease (CKD) [2, 19, 20].

All individuals with T2DM should be screened for CKD using the spot urine albumin-to-creatinine ratio and estimated glomerular filtration rate at the time of diagnosis and at least once a year after that. Although the exact cause of chronic kidney disease (CKD) is still unknown, research has shown that a number of risk factors, including obesity, ageing, hypertension, diabetes mellitus, hyperlipidemia, use of nephrotoxic medications, family history of kidney disease, smoking, heavy drinking, HIV infection, electrolyte and acid-base disturbances, low-income occupation, use of traditional medication, low hemoglobin, rapid urbanization, physical inactivity, and rapid population growth [21–25].

The study in Japan revealed that elderly patients with type 2 diabetes, renal dysfunction is characterized by low eGFR [26]. One of the main challenges of diagnosing CKD in diabetic patients is that it is asymptomatic onset with reduced quality of life, high morbidity and mortality rate [2, 27].

CKD is estimated to affect 50% patients with T2DM globally, and its presence and severity markedly influences disease prognosis. CKD is more common in certain patient populations, including the elderly, those with youth-onset diabetes mellitus, those who are obese, certain ethnic groups, and disadvantaged populations. The incident cases of CKD as a result of type 2 diabetes mellitus worldwide in 2017 had increased by 74% compared with 1990 [28–30].The study showed that prevalence of CKD ranged from 11 to 90% in patients with diabetes, high-risk groups in patients with HIV from 1–46%,and 13–51% in patients with hypertension [31].

In order to improve patients’ quality of life, early screening for CKD is crucial for diagnosis and treatment. Other interventions include counselling, good glycemic and blood pressure control, and medication use. Renin-angiotensin system inhibitors, sodium-glucose cotransporter-2 inhibitors, glucagon-like peptide 1 receptor agonists, and, more recently, non-steroidal mineralocorticoid receptor antagonists are among the medications that have been demonstrated to slow the progression of CKD [24, 32–34].

Diabetes is the leading cause of CKD in all developed and most developing countries, with approximately 20% of people with type 2 diabetes showing evidence of diabetic nephropathy within 20 years of diabetes onset [35]. The patients with diabetes are likely to be prescribed more than one type of medication which exposed them at a higher risk of CKD due to the effect of polypharmacy [36, 37]. Studies showed that early detection and treatment of diabetes and chronic diseases can slow or prevent the progression of CKD [38] .This study aimed to estimate the global prevalence of CKD and its associated factors among type 2 diabetes patients that can provides a scientific evidence for a better understanding of the burden of CKD among type 2 diabetes mellitus patients and design interventional strategies.

## Methodology

### Search strategy

This systematic review and meta-analysis was performed by following Preferred Reporting Items for Systematic Reviews and Meta-Analyses (PRISMA) guidelines [39]. A search strategy was implemented using electronic databases (PubMed, Cochrane Library, Google Scholar, and grey literature), which were systematically searched online to retrieve related articles. The literature search was conducted by using the key words “chronic kidney disease” OR (chronic renal failure) AND “Type 2 diabetes mellitus” OR “T2DM starting from May 30, 2018 to May 30, 2023 were included. The retrieved studies references were also screened and checked. The review protocol is available on PROSPERO (ID: CRD42023433892).

### Eligibility criteria

For the review, CoCoPop mnemonic (Condition, Context and Population) approach was used. Studies that reported chronic kidney disease prevalence and associated factors among type 2 diabetic patients globally with any type of study design at any health facility level, studies with open or free access to full text and written in English language were included. The studies without abstract & full-text, reports, and qualitative studies, conference summaries were excluded. Articles were assessed for inclusion using their title, abstract and then a full review of articles was done before included to the final review.

### Data extraction and management

Eligible studies were imported to Endnote v.9 and duplicates were removed. The two independent reviewers (TFA, EKB,) were do the abstract and full text review and data abstraction. Any disagreements were resolved by consensus and involvement of third reviewer (ETF). Full text articles were retrieved for studies that meet the inclusion criteria. Data extraction used a data collection form and was performed by two blind and independent reviewers. The following data were extracted: author, publication year, study design, place of study, sample size, participants.

## Quality assessment

The Joanna Briggs Institute (JBI) critical appraisal check list was used to assess the quality of studies which is freely available at https://jbi.global/critical-appraisal-tools. Using the tool as a protocol, the reviewers (NK, SSG) used the blinded review approach to evaluate the quality of the original articles. Those studies, scores 5 or more in JBI criteria were considered to have good quality and included in the review [40]. Discrepancies in the quality assessment was resolved through a third author (EMM).

## Statistical analysis

The necessary data were extracted from the studies using Microsoft Excel V.2016 and the extracted data were exported to STATA version 17 software for analysis. The articles were summarized by tables and forest plot. The standard error and 95% confidence interva1 for the prevalence of chronic kidney disease was calculated for those studies in which estimates of standard error and 95% confidence interval for the prevalence of chronic kidney disease were not found in their full text of their article.

Meta-analysis was performed, the significance of the pooled Odds Ratio (OR) was determined by Z-test. The pooled estimate for the prevalence of CKD in patients with diabetes was calculated using random-effect models with 95% confidence intervals (CIs). The statistical heterogeneity was checked subjectively by using forest plot, and objectively by Cochrane Q-test and _I_^2^ statistics [41]. The presence of publication bias was checked by using a funnel plot and Egger’s and Begg’s statistical tests [42].

## Results

**Fig. 1:**
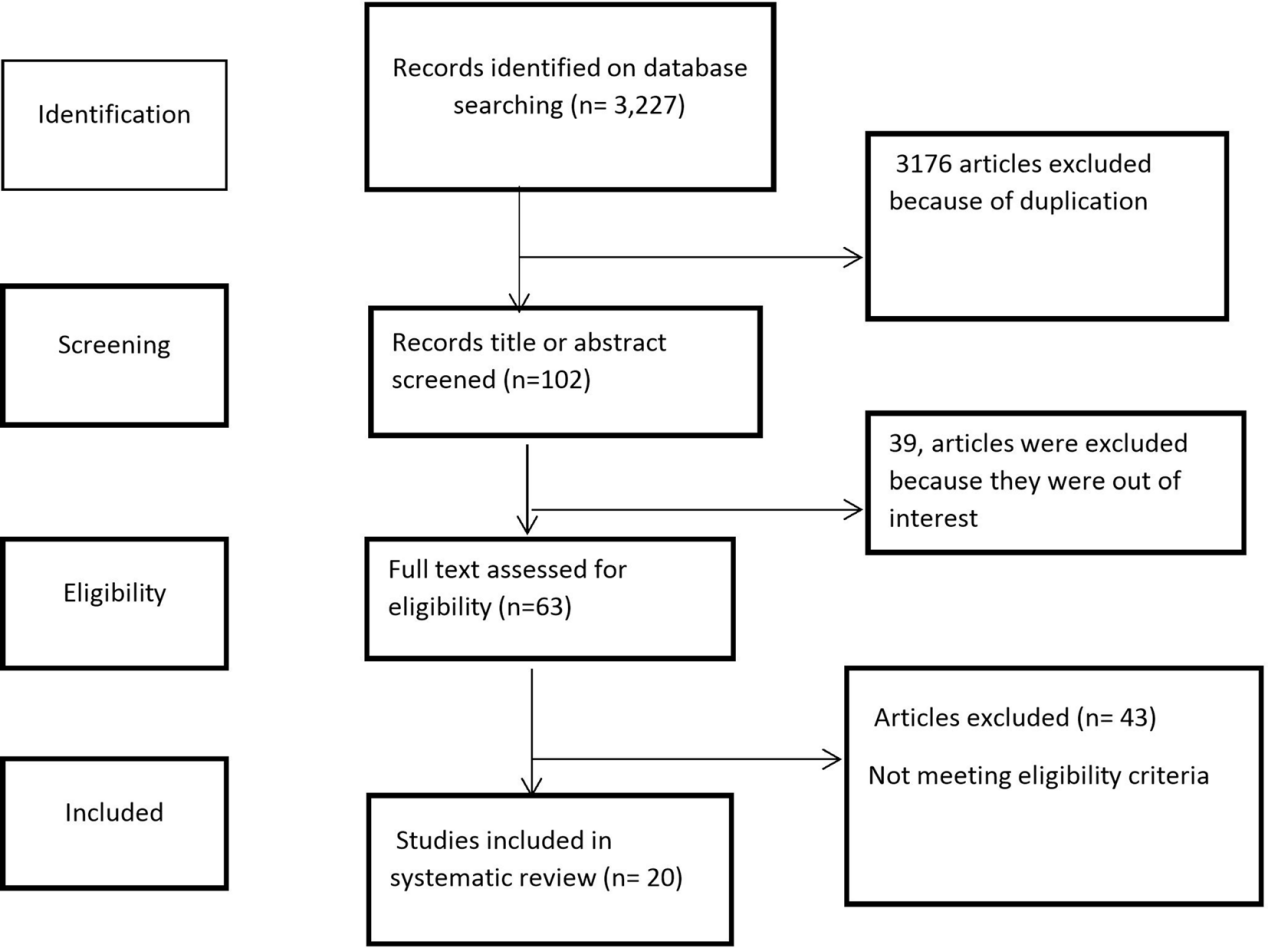
PRISMA flow diagram of study selection

In this systematic review and meta-analysis, a total of three thousand two hundred seventy-eight (3,278) record articles were searched from all searched database sources. After removing duplicates (3176), reviewing title and abstract (102), and removal of 39 articles due to not reporting for prevalence of chronic disease among diabetes mellitus type 2 patients, then 63 articles were eligible for full text review and finally 20 articles included for systematic review and meta-analysis (Fig. 1).

### Study characteristics

In this systematic review and meta-analysis 20 studies were done in 13 different countries globally. Two studies done in Ethiopia [43, 44],one study each in Ghana [45], United Arab Emirates [46], Australia [47], Germen [48], united kingdom, Malaysia, Palestine [49–51], three studies in USA [52–54] ,two in China [55, 56],two in Spain [57, 58], three in Thailand [59–61], and in six countries in Europe and Asia [62].in this study most of the studies were hospital based except two are community level. The total sample size was 1,711,926 with maximum sample 1, 177 896 sample in six European and Asian countries [62] and minimum of 119 sample size in Ethiopia [44] (Table 1).

**Table 1:** Characteristics of studies included in the systematic review and meta-analysis of the magnitude of chronic kidney disease among type 2 Dm patients worldwide

Authors/ publication year	Region	Study design	Study setting	Sample size	Prevalence of CKD (%)	Quality assessment based on JBI
Anunya Pradidthaprecha, et,al (2021)[50]	Thailand	retrospective	hospital based	3,465	29.70%	9
Junlin Zhang etal(2022)[39]	USA	retrospective	hospital based	2720	35.06%	8
Janjira Jitraknatee etal/2020[49]	Thailand	cross-sectional	primary care	1,096	24.40%	8
Sojib Bin Zaman etal/2018[51]	Thailand	retrospective	district hospital	3,580	23.26%	6
Ruth Usó-Talamantes etal.2021[48]	Spain	cross-sectional	Spanish Health District	14,935	33.80%	9
Juan,Martínez Candelaa.etal/2018[47]	Spain	cross-sectional	Primary Care	939	37.20%	8
Zaher Nazzal.etal/2020[42]	Palestine	cross-sectional	PHC clinics	385	23.60%	7
Ruth Sim etal./2023 [41]	malasiya	cross-sectional	two tertiary hospitals	1992	15%	6
Kåre I. Birkeland MD etal/2020[52]	Europe and Asia	cross-sectional	population-based study	1, 177 896	36%	6
Medina Abdela Ahmed etal/2022[33]	Ethiopia	retrospective	Referral hospitals	415	10.80%	8
Petter Bjornstad, M.D.,etal/2021[43]	USA	prospective	Hospital based	500	54.80%	6
S Al-Shamsi et al. /2018[36]	United Arab Emirates	retrospective	two public hospitals	251	37.70%	7
Lin Yang etal/2018[46]	China	prospective	primary care setting	31,574	29.70%	9
Jiayu Duan1etal/2019[45]	China	prospective	rural districts	2710	35.50%	
Shewaneh Damtie1etal/2018[34]	Ethiopia	cross-sectional	hospital based	119	3.751	8
Elliot K. Tannor, MD et al. /2019[35]	Ghana	cross-sectional	hospital based	348	16.10%	8
Antonio González-Pérez etal/2019[40]	UK	Cohort	hospital based	109,365	26%	7
Louisa Sukkar etal/2020[37]	Australia	prospective	population-based	9,313	22.60%	6
Peter Bramlage1 et al. 2019[38]	Germany	cross-sectional	hospital based	343,675	50%	9
Darren K. McGuire MD etal/ 2023 [44]	USA	randomized controlled	hospital based	9650	42.3%	7

### Meta -analysis

#### Pooled prevalence of chronic Kidney Disease among type 2 DM patients

**Fig. 2:**
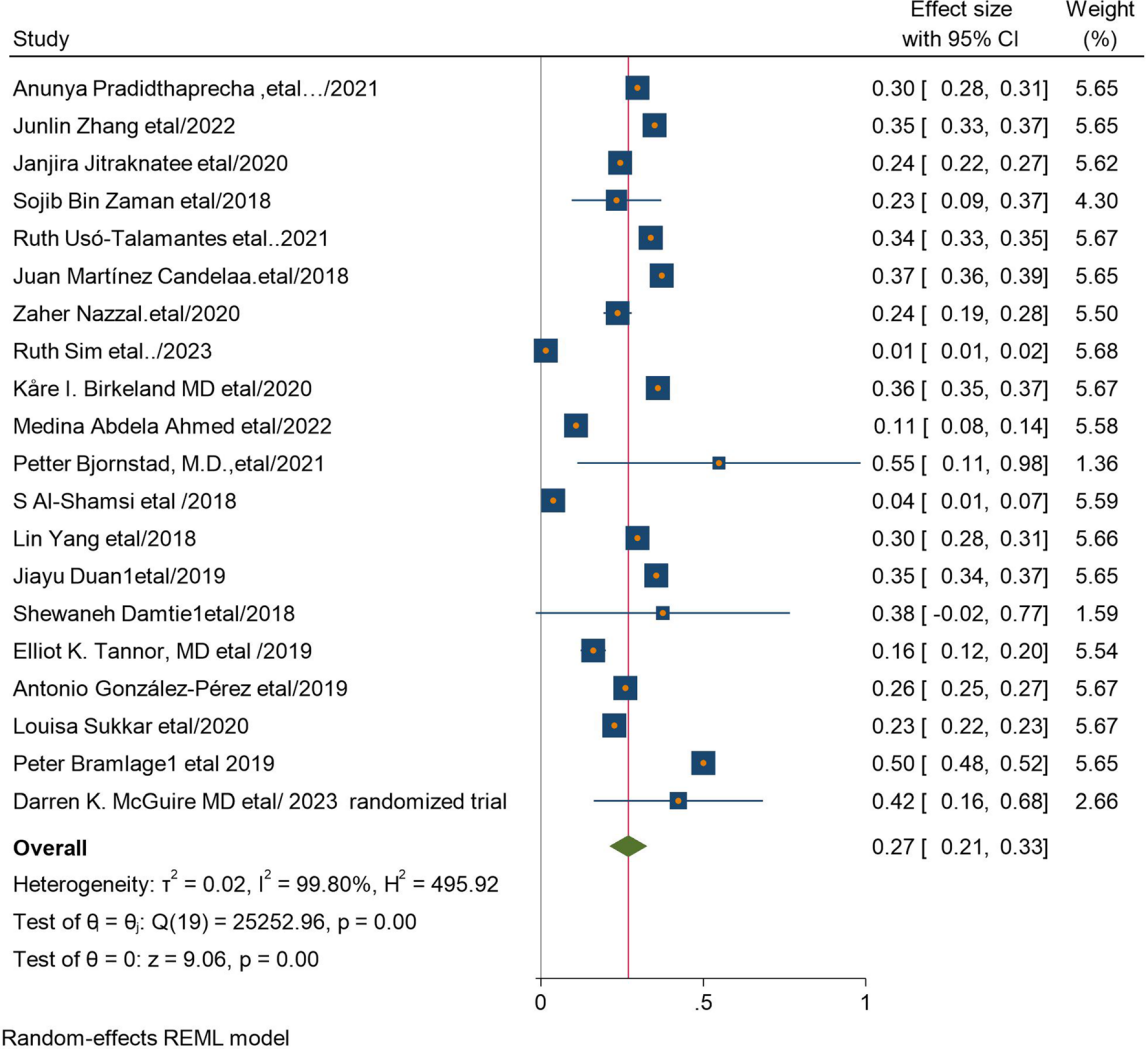
Forest plot of the pooled magnitude of CKD among type 2 diabetes mellites patients

The pooled magnitude chronic kidney disease among type 2 DM patients was 27% (95% CI 21%, 33%). Based on tau square (between studies variance), tau2 = 25252.96 & I2 = 99.8 with p value < 0.00005 which indicates there is statistically significant heterogeneity among studies (Fig. 2).

### Publication bias

**Fig. 3:**
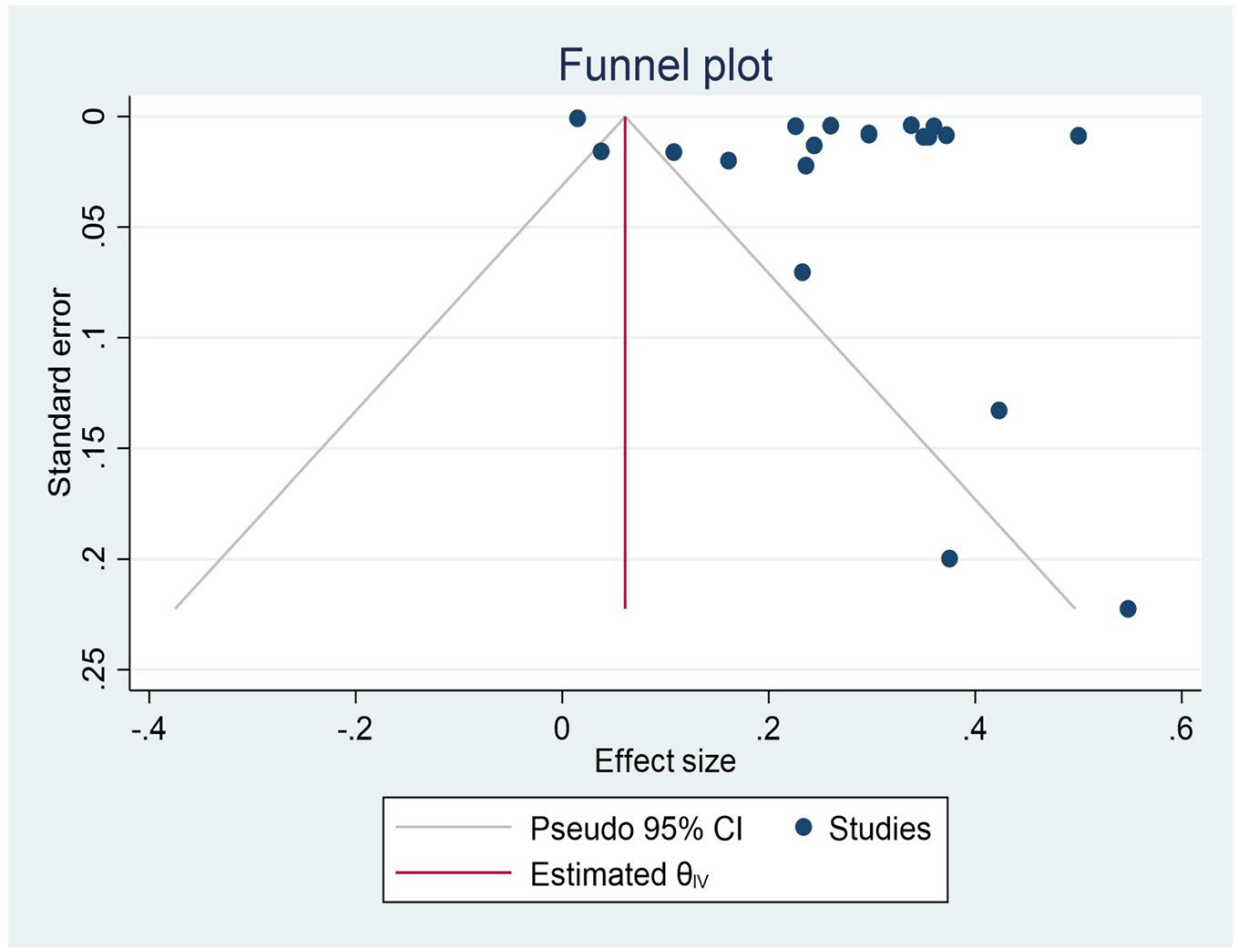
A funnel plot displaying publication bias across the studies

The result of regression-based egger test for small-study effects showed significant result (p value = 0.2540). The funnel plot’s evidence suggests the current study had a source of publication bias. The results seemed like asymmetrical funnel plots. The result of egger’s test was not statistically significant for the presence of publication bias (P = 0.254). and Begg’s test for small-study effects was significant (p = 0.0012) (Fig. 3).

### Factors associated with CKD among type 2 diabetic patients

**Fig. 4:**
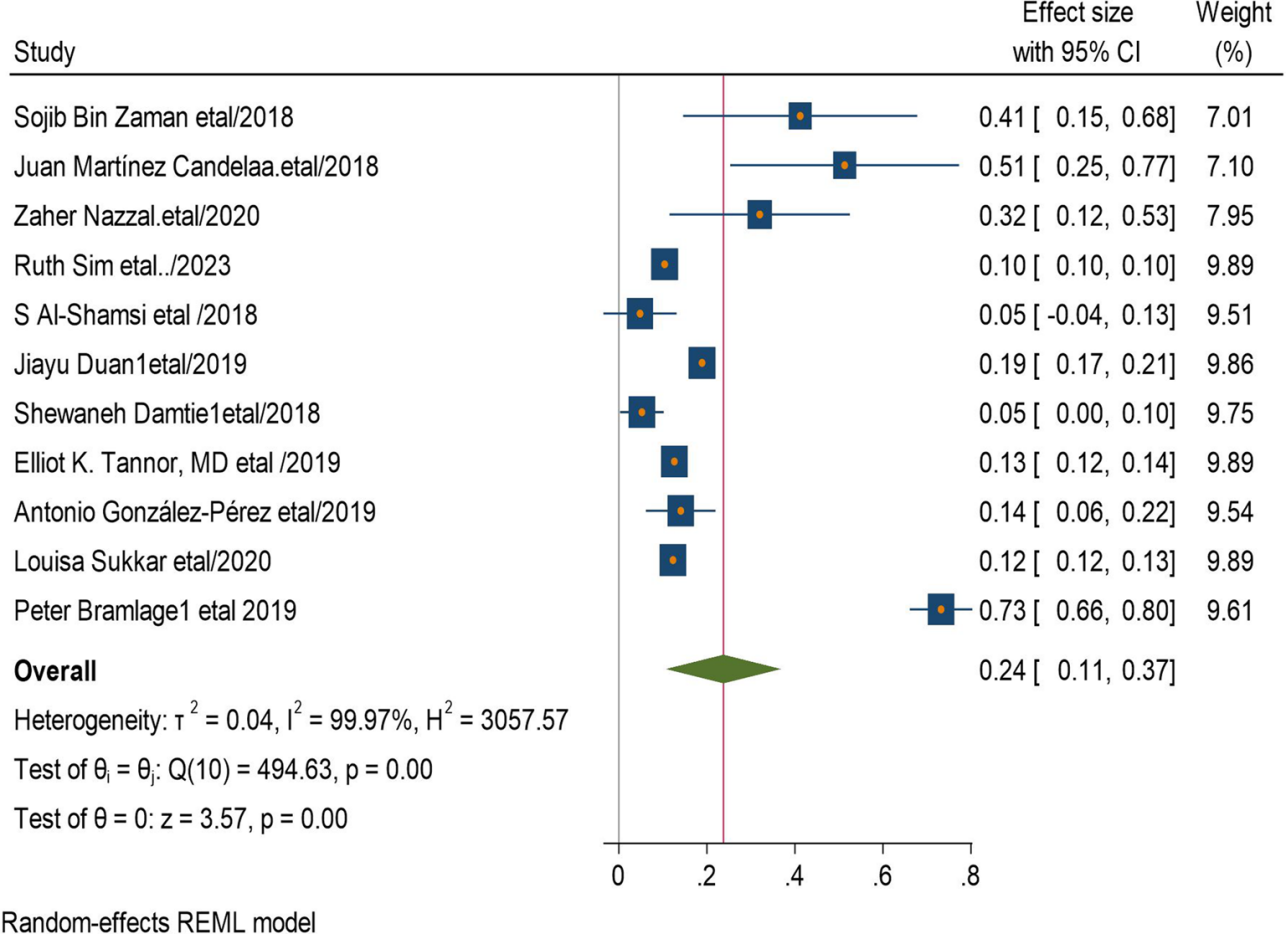
Association between age and chronic kidney disease among type 2 DM patients

**Fig. 5:**
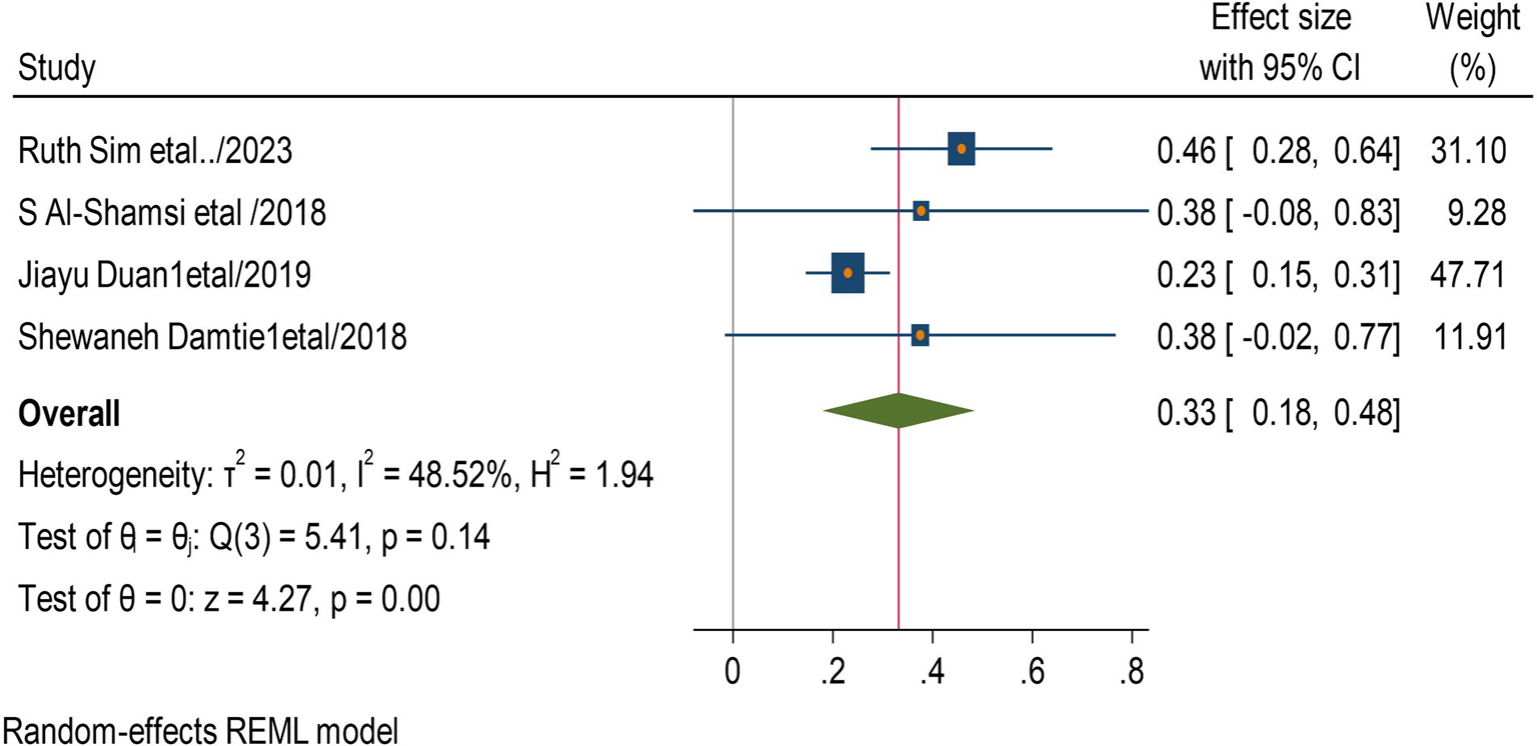
Association between history of type 2 DM and chronic kidney disease among type 2 DM patients

In this systematic review and meta-analysis increased age, obesity, duration of diabetes mellitus, smoking history, presence of hypertension, and having cardiac heart disease were factors significantly associated with the presence chronic kidney disease among type 2 diabetic patients. Eleven studies had shown the association between increased age and he development of CKD among type 2 diabetic patients. The pooled odds ratio showed that diabetics patients with increased age were 24 times more likely to develop CKD than those with younger (AOR = 24, 95 CI, (0.11–0.37) (Fig. 4). Four studies revealed that having history of type 2 diabetics has 33 times increased risk of CKD than non-diabetics patients (AOR = 33,95 CI, (0.18–0.48) (Fig. 5).

**Fig. 6:**
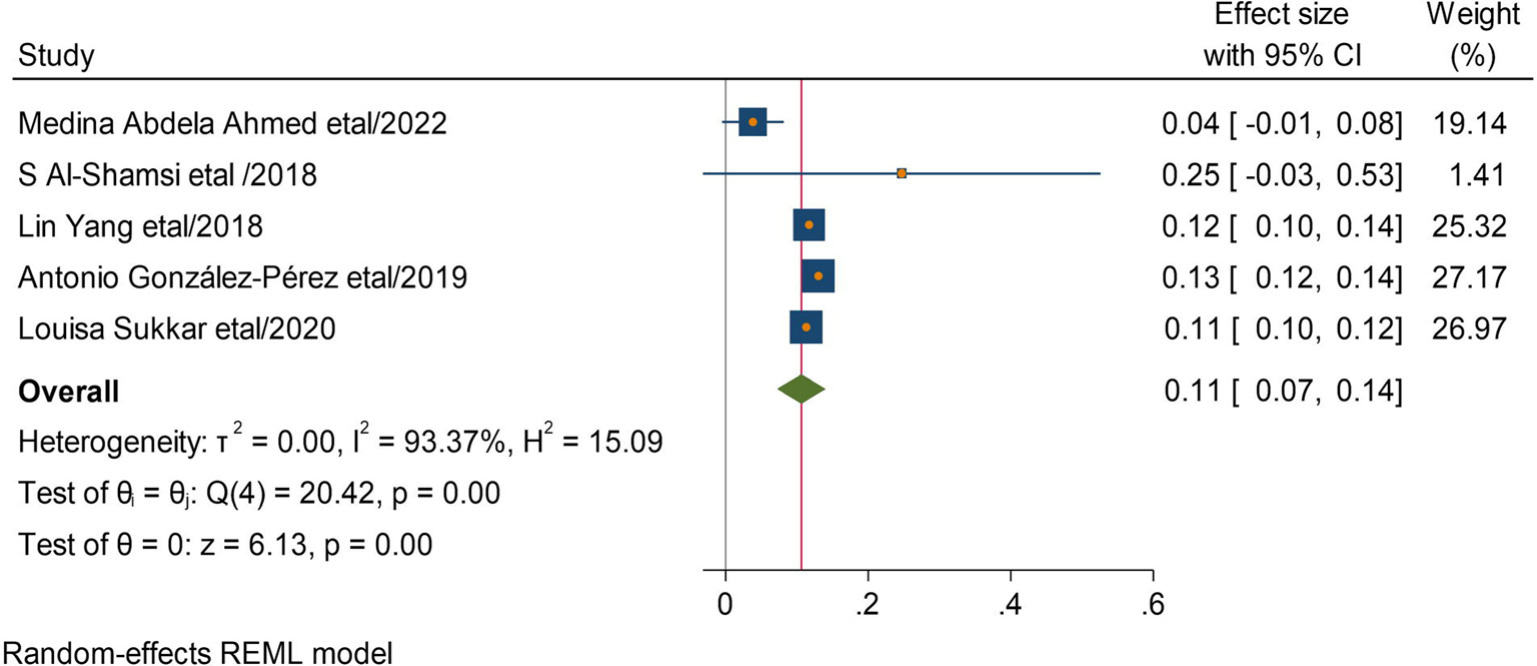
Association between having cardiac and chronic kidney disease among type 2 DM patients

**Fig. 7:**
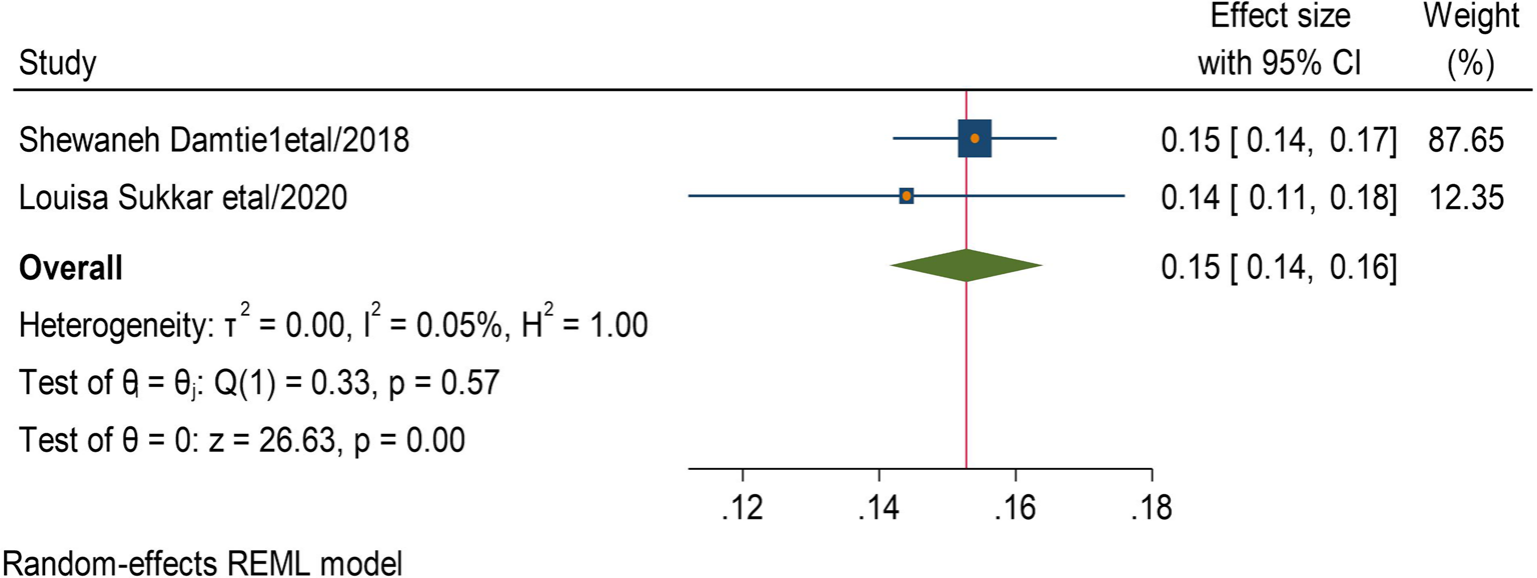
Association between obesity and chronic kidney disease among type 2 DM patients

Five studies reported that having cardiac disease with DM has 11 times More likely to develop CKD than without. (AOR = 11,95 CI, (0.7 − 0.14) (Fig. 6). Two studies showed that being obesity has 15 times more likely CKF than normal. AOR = 15,95 CI, (0.14–0.16)) (Fig. 7).

**Fig. 8:**
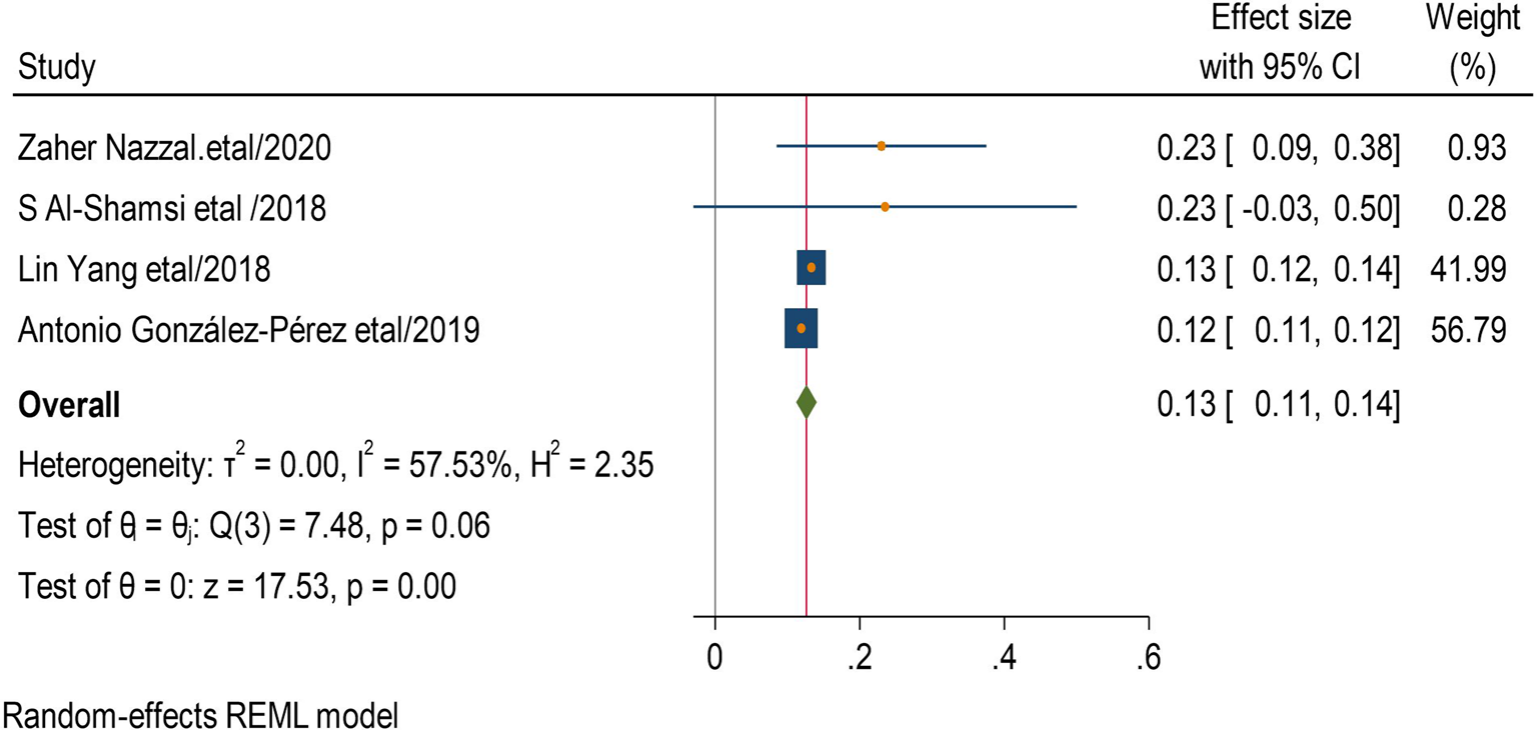
Association between smoking and chronic kidney disease among type 2 DM patients

**Fig. 9:**
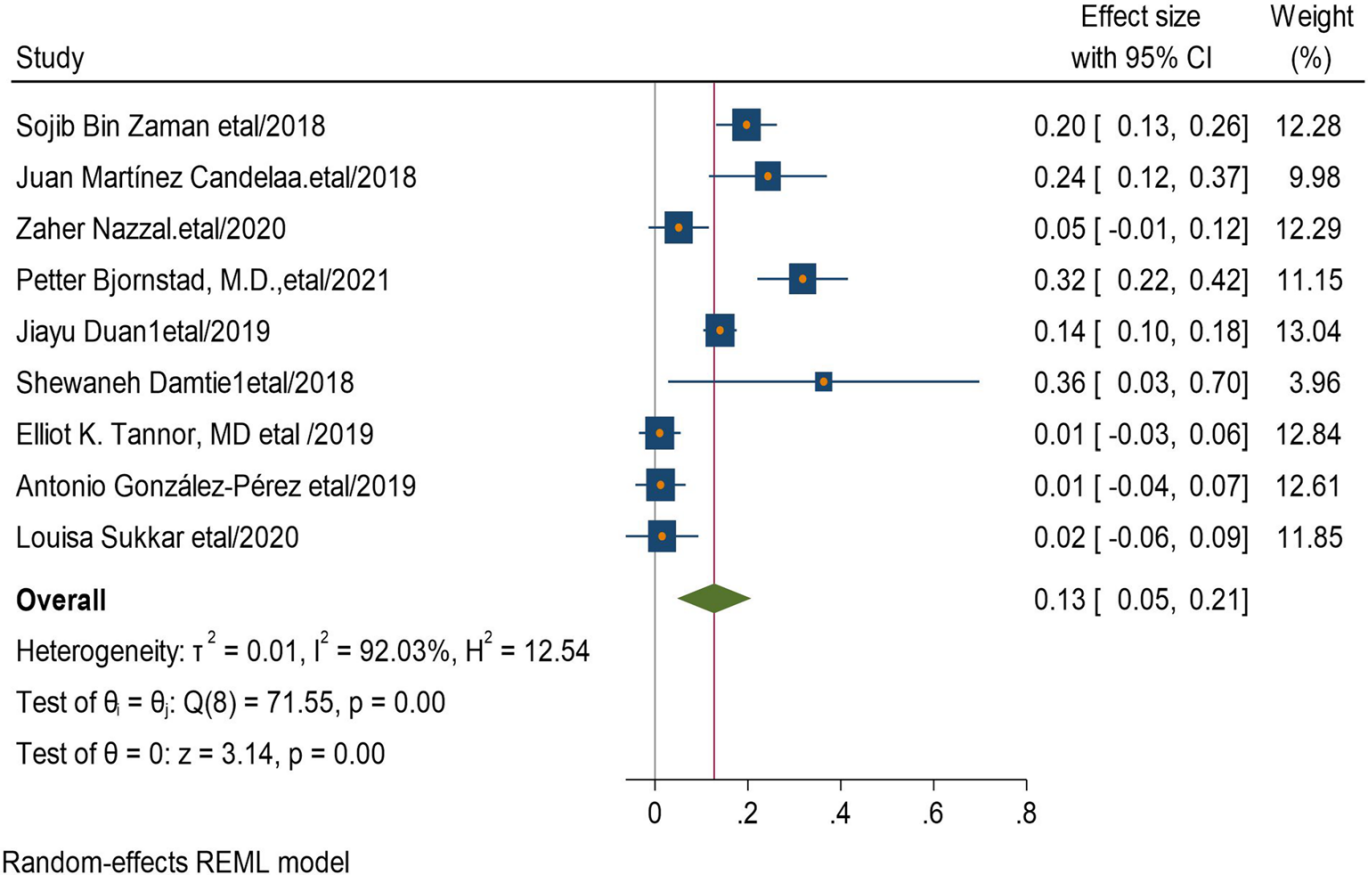
Association between hypertension and chronic kidney disease among type 2 DM patients

Four studies describe that smokers were 13 times more likely to develop CKD than non-smokers (AOR = 13,95 CI, (0.11–0.14) (Fig. 8). Nine studies discussed that having hypertension was 13 times more likely to get CKD than non-hypertensive DM patients (AOR = 13,95 CI, (0.05–0.21) (Fig. 9).

## Discussion

Chronic kidney disease is associated with several consequences, including anemia, bone and mineral disorders, electrolyte imbalance, acid-base abnormalities, sexual dysfunction, hypertension, cardiovascular diseases [63]. Globally 850 million individuals live with the chronic kidney disease with all-age prevalence of 29.3% [64, 65]. Type 2 diabetes, hypertension, and glomerulonephritis are the most frequent underlying diseases that lead to the development of CKD. Patients with CKD have an elevated risk of severe renal and cardiovascular morbidity and mortality [66–68].

Among the drugs that have been shown to slow the progression of chronic kidney disease are glucagon-like peptide 1 receptor agonists, sodium-glucose cotransporter-2 inhibitors, renin-angiotensin system inhibitors, and, more recently, non-steroidal mineralocorticoid receptor antagonists. Neither an ACE inhibitor nor an angiotensin receptor blocker is recommended for the primary prevention of chronic kidney disease in diabetics with normal blood pressure, urine albumin-to-creatinine ratio (< 30 mg/g creatinine), and normal estimated glomerular filtration rate. For people with type 2 diabetes and diabetic kidney disease, it is recommended to use a sodium-glucose cotransporter 2 inhibitor to slow the progression of cardiovascular events and chronic kidney disease in patients with estimated glomerular filtration rate ≥ 20 mL/min/1.73 m2 and urinary albumin ≥ 200 mg/g creatinine [18, 36, 69].

End-stage renal disease (ESRD) resulting from diabetic kidney disease has a number of systemic effects that lower quality of life, including electrolyte abnormalities, problems with bone metabolism, and renal anemia. Comprehensive care of hyperglycemia, hypertension, dyslipidemia, and healthy lifestyle choices is typically provided to those with diabetes and chronic kidney disease [70–72]. Approximately 40% of those with type 2 diabetes will develop CKD, and the risk rises with the duration of the disease. When compared to costs for people with diabetes alone, having CKD results in an average increase in healthcare costs of over 50% [73, 74].

The goal of this systematic review and meta-analysis was to generate pooled estimates of the prevalence of chronic kidney disease among individuals who have type 2 diabetes mellitus globally. Based on the review the prevalence of chronic kidney disease among type 2 diabetes mellites was 27% (95% CI 21%, 33%).This was comparable with systematic review and meta-analysis study in Africa, which reported 24.7% pooled prevalence of CKD [75],in the middle east region the prevalence of CKD was 28.96% [76], Iran prevalence of nephropathy in patients with type 2 diabetes was 30.6% [77], in Asia the prevalence of CKD was 31% in adults with DM [78], and another systematic review and meta-analysis which include cohort studies in Europe, Asia and America report the prevalence of CKD among T2DM was approximately 29.1% [79].

This systematic review and meta-analysis was lower than study reported a pooled prevalence of end stage renal disease was 73% [80] and in sub Saharan Africa the overall prevalence of diabetic nephropathy was 35.3% [81] and the study was higher than the study in Africa among the general population which was 15.8% [82], In Asia the pooled prevalence of CKD among the general population was 14% [78],another cohort systematic review and meta-analysis reported that ESRD among on T2DM was 1.1% [83]. The discrepancy could be differences in study period, sample size, demographics characteristics, diabetic duration, and study design of included studies.

This systematic review and meta-analysis include 20 studies in 13 countries with total 1,711,926 study participants. According to the review, USA had the highest incidence of CKD at 54.8% and the United Arab Emirates had the lowest prevalence at 4% [46, 52]. The funnel plot’s evidence the results seemed like asymmetrical funnel plots suggests current study had a source of publication bias. but the result of egger’s test was not statistically significant for the presence of publication bias (P = 0.254).

The study revealed that being old age, hypertension, cardiac disease, smoking, obesity, having type 2 diabetes mellitus was predictor variable for presence of chronic kidney disease among type 2 diabetic patients. This was consistent with the study reported that age, smoking, and DM duration has significant association with CKD development among diabetic patient [83].Another systematic review showed that duration of diabetics and having hypertension and obesity has increased risk of chronic kidney disease among diabetic patients [81, 84, 85]. The study in China also showed that old age, smoking, and hypertension were predictor variables of CKD with increased morbidity and mortality in T2DM patients [86].

## Conclusion

This systematic review and meta-analysis revealed that the prevalence of chronic kidney disease among type 2 diabetes mellites patients was high based on the included 20 articles. The prevalence of chronic kidney disease varies across country with the highest in USA and the lowest in Ethiopia. The review reported that old age, hypertension, having cardiac disease, smoking, obesity, having history of type 2 diabetes mellitus were predictor variables for chronic kidney disease among diabetic patients. Patients with CKD have an elevated risk of severe renal and cardiovascular morbidity and mortality. Therefore, in order to lower the morbidity and mortality from chronic kidney disease among type 2 diabetic patients, it is advised to develop both preventive and curative intervention strategies, such as raising awareness, creating a supportive environment and prescribing appropriate medication at an early stage.

## Declarations

### Data Availability

The datasets used and analyzed during the current study are available from the corresponding author on reasonable request.
